# Evaluation of a disposable stirred tank bioreactor for cultivation of mammalian cells

**DOI:** 10.1186/1753-6561-5-S8-P54

**Published:** 2011-11-22

**Authors:** Alexander Hähnel, Benjamin Pütz, Kai Iding, Tabea Niediek, Frank Gudermann, Dirk Lütkemeyer

**Affiliations:** 1 Institute for Protein Characterisation, Faculty of Engineering and Mathematics, University of Applied Sciences, Bielefeld, Germany; 2 BIBITEC GmbH, Bielefeld, Germany; 3 Institute of Technical Analytics, Faculty of Engineering and Mathematics, University of Applied Sciences, Bielefeld, Germany

## Introduction

Disposable bioreactors are increasingly gaining acceptance for cell culture applications due to a number of advantages including ease of use and reduced labour costs, less requirements for utilities such as steam and purified water. In addition, the system requires no cleaning or cleaning validation and only a reduced clean room footprint. Integrated ready to use pre-sterilised disposable sensors for pH and dO_2_ monitoring are expected to reduce the risk of contamination compared to conventional electrodes. Accordingly, a disposable pre-sterilised and therefore ready-to-use 200 L bioreactor featuring optical sensors for dO_2_ and pH has been evaluated for suspension culture of CHO cells in protein free media.

## Materials and methods

Several fed-batch cultivations with a maximum cultivation time of 9 days were performed as follows.

Cells: Recombinant CHO cell lines producing monoclonal antibodies

Media: MAM-PF2, Bioconcept, Allschwil, Switzerland

Feed and medium from Teutocell, Bielefeld, Germany

Both media are chemically defined and protein free.

Feeds: Different commercial available amino acid concentrates, glucose and glutamine

Bioreactor: BIOSTAT® CultiBag STR 200 L, Sartorius Stedim biotech, Göttingen, Germany

Parameter control: Agitation of the culture was performed by a pre-installed magnetic driven stirrer with two 3-blade impellers at 80 rpm, pH-adjustment via adding CO_2_ to the overlay stream and dO_2_ was controlled via aeration of pure oxygen through the ring-sparger.

## Results

**Figure 1:**
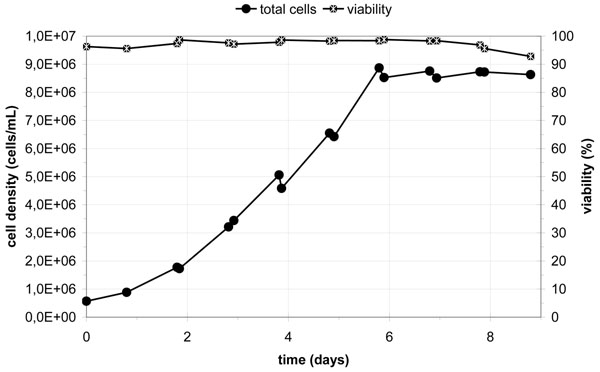
Cell growth and viability during a fed-batch cultivation of CHO cells in the 200 L disposable bioreactor.

Maximum cell densities between 5.0 x 10^6^ and 1.3 x 10^7^ cells/mL with viabilities between 85 % and 100 % were achieved. Results of one typical fed-batch cultivation are shown in figure 1.

The cell densities depended more on the medium performance and feeding strategy than on the bioreactor-features itself. At a cell density of 1.3 x 10^7^ cells/mL the volume flow of pure oxygen was at a rate of 0.85 L/min. In regard to the system’s maximum flow rate of 20 L/min, the throughput of oxygen most likely would not become a bottleneck of even higher cell densities. Detailed comparison of the optical pH and dO_2_ probes with their conventional counterparts revealed a reliable concordant performance.

## Conclusion

The disposable BIOSTAT® CultiBag STR 200 L bioreactor is a reliable cultivation system for high cell density operations above 10^7^ cells/mL with mammalian cells. The usually installed ring-sparger supplies a sufficient amount of gas into the liquid phase and still offers adequate reserves. In direct comparison the optical sensors correlated excellently with the conventional electrodes. External pH-testing and recalibration is mandatory to compensate for the drift of the pH-sensor. The reactor’s overall performance is convincing in this field of operation, which is mainly due to its ease of use combined with the low utilisation of space. The set up procedure can be done in less than three hours which is remarkable compared with stainless steel bio-reactors and the advantage in prevention of cross-contaminants during changeover of campaigns is preeminent.

